# Augmenting visual feedback with visualized interaction forces in haptic-assisted virtual-reality teleoperation

**DOI:** 10.3389/frobt.2024.1427095

**Published:** 2024-12-18

**Authors:** Alex van den Berg, Jelle Hofland, Cock J. M. Heemskerk, David A. Abbink, Luka Peternel

**Affiliations:** ^1^ Department of Cognitive Robotics, Delft University of Technology, Delft, Netherlands; ^2^ Heemskerk Innovative Technology B.V., Delft, Netherlands

**Keywords:** teleoperation, visual cues, virtual reality, head-mounted display, force feedback, virtual fixtures

## Abstract

In recent years, providing additional visual feedback about the interaction forces has been found to offer benefits to haptic-assisted teleoperation. However, there is limited insight into the effects of the design of force feedback-related visual cues and the type of visual display on the performance of teleoperation of robotic arms executing industrial tasks. In this study, we provide new insights into this interaction by extending these findings to the haptic assistance teleoperation of a simulated robotic arm in a virtual environment, in which the haptic assistance is comprised of a set of virtual fixtures. We design a novel method for providing visual cues about the interaction forces to complement the haptic assistance and augment visual feedback in virtual reality with a head-mounted display. We evaluate the visual cues method and head-mounted display method through human factors experiments in a teleoperated dross removal use case. The results show that both methods are beneficial for task performance, each of them having stronger points in different aspects of the operation. The visual cues method was found to significantly improve safety in terms of peak collision force, whereas the head-mounted display additionally improves the performance significantly. Furthermore, positive scores of the subjective analysis indicate an increased user acceptance of both methods. This work provides a new study on the importance of visual feedback related to (interaction) forces and spatial information for haptic assistance and provides two methods to take advantage of its potential benefits in the teleoperation of robotic arms.

## 1 Introduction

Haptic assistance (HA) is a promising compromise between manual teleoperation and full automation (Abbink et al., 2012). In HA, guidance forces are provided to the operator using a mechanical haptic interface to combine human intelligence and creativity with the benefits of automation systems. Despite the benefits of HA, some issues in human-automation interaction remain unsolved (Boessenkool et al., 2013). One of the key potential problems is that either the human does not understand the automation, or the automation does not understand the human (Abbink et al., 2012). This mismatch in understanding has been found in several HA interfaces and is thought to be a major cause of a low user acceptance (Borst et al., 2013; Nakazawa et al., 2016).

As the degree of automation increases, it becomes critical that the human operator has access to information about what the automated agents are doing and what they will be doing next (Christoffersen and Woods, 2002). Nevertheless, the complexity and amount of information that can be shared through haptic forces is limited. Consequently, as more information is being conveyed through these haptic forces, the information may become ambiguous and difficult to interpret, thus potentially resulting in the problems stated above. Furthermore, in teleoperation, the human operator needs to adaptively control physical interaction between a remote robot and the environment to successfully perform complex tasks (Peternel and Ajoudani, 2022). Therefore, rich feedback about interaction forces is essential.

To increase the complexity and amount of information about interaction forces, visual feedback can augment HA. Two key factors in such an interface are the specific design of visual cues and the type of display that is used for providing the cues to the operator. In terms of the type of display, a head-mounted display (HMD) is a promising alternative to standard desktop monitors since HMD can provide richer information to the operator (Ragan et al., 2012). Additionally, HMDs can increase the sense of immersion, which can improve teleoperation performance and user acceptance (Liu et al., 2017; Xi et al., 2019; Whitney et al., 2020). Finally, HMD is often employed to display virtual reality (VR), which is an important element for operator training systems (Jourdes et al., 2022; Sunesson et al., 2023; Kuitert et al., 2023).

In this paper, we focus on a human factors study of teleoperated robotic arms in VR using HMD and visual feedback about interaction forces. There is some important related work to consider in that direction. The study in (Arevalo Arboleda et al., 2021) displayed visual cues through HMD to help orient the robotic arm gripper for object grasping during a pick-and-place task. In Chan et al. (2022), HMD and VR were used to give visual cues about the key movement waypoints required for a teleoperated pleating task. However, the visual cues in those two studies were limited to movements, and no information about interaction forces was displayed either haptically or visually. The study in Kuitert et al. (2023) developed a novel workspace visualization method for HDM and analyzed its effects in combination with haptic feedback during a welding task. In Girbés-Juan et al. (2020) haptic feedback was studied in combination with HMD-based VR feedback in object handling and wiping tasks in dual-arm teleoperation. Nevertheless, no visual feedback for interaction forces was provided in those two studies and the operator had to rely on haptic cues. The study in Clemente et al. (2016) provided visual cues about the grip force and gripper closure that were displayed through HMD and VR for teleoperated object grasping. Nevertheless, operators did not receive haptic feedback at the hand as the robot was operated by a sensorized glove rather than a haptic device. Furthermore, visual cues were limited to grip forces as opposed to direct interaction forces. The method in Jourdes et al. (2022) provided visual feedback about interaction forces during teleoperated robotic suturing but did not use HMD or perform any human factor experiments to analyze usability. To the best of our knowledge, the design and effects of additional visual cues related to complex interaction forces between the robot tool and environment (i.e., in addition to haptic force feedback) using HMD during the teleoperation of robotic arms in VR have not yet been investigated.

To fill in this gap, we design a novel method for providing visual cues about the interaction forces to complement HA and study their effects during the teleoperation of a simulated robotic arm for a dross removal use case in VR. Furthermore, we examine the effects of displaying the visual cues about the interaction forces with HMD compared to a desktop monitor. Both display methods, and the interaction between them, are evaluated in a two-way human factors experiment, performed on a real-hardware haptic robot device that teleoperates a simulated robotic arm during dross removal in a virtual environment. The experimental evaluation aims to provide new insights into the importance of visual feedback design in HA teleoperation, and to provide a set of recommendations regarding their applicability for the proposed and similar use cases.

The main contributions of this paper are: 1) a novel method for providing visual cues about the interaction forces, 2) a study and analysis of the effects of providing additional visual cues about the interaction forces, 3) a study and analysis of the display method provided either by HMD-based VR method or standard monitor, 4), a study of interaction effects between the two factors (i.e., visual cues and type of display method), all specific to the teleoperation of a simulated robotic arm performing a dross removal task in a remote environment. The resulting novel knowledge can be used as a guideline for the design of HA teleoperation systems for similar manufacturing tasks, or for the development of operator training systems.

Based on related work, we hypothesize the following:

• H1: The proposed visual cues method helps human operators to improve *task performance* in industrial tasks such as dross removal.
• H2: The proposed visual cues method helps human operators to increase *user acceptance.*
• H3: The proposed visual cues method helps human operators to increase *safety.*
• H4: The use of the HMD-based VR instead of the desktop monitor helps to improve *task performance.*
• H5: The use of the HMD-based VR instead of the desktop monitor helps to improve *user acceptance.*
• H6: There is no interaction between these factors, or in other words, the hypothesized improvements are present regardless of the state of the other factor.

The rationale behind H6 is that even though the information presented by the visual cues method and the HMD method partially overlap (both provide additional depth perception), each of the methods has its benefits. The main additional benefit of visual cues is that they provide information about the forces and the VFs, whereas the HMD method provides an increased sense of immersion.

In addition to the main analysis, we also conducted a supplementary analysis with four more metrics related to task performance of dross removal tasks: submergence rate, movement velocity, number of scooping actions, and scoop size. These give some further insights into the effects of HMD and visual cues on the teleoperated task with simulated robotic arms in a virtual remote environment.

## 2 System design

### 2.1 Use case and hardware

The use case that was chosen for this study is the removal of dross from a zinc bath in a continuous galvanizing line (CGL). Dross is floating solid contamination in the zinc bath that needs to be removed to maintain a high-quality coating (Porter, 1991). Its removal is a labor-intensive job with poor work conditions, potential safety hazards, and difficulties in controlling operating costs and quality (Hao et al., 2018). Furthermore, the environment is subject to changes, e.g., the liquid metal is solidifying on the tools and the environment, and there is a fluctuation of liquid level in the zinc bath. Additionally, failing to perform this dross removal operation adequately could result in significant financial losses. Current robotic dross removal solutions rely on rigid automation systems. For example, the robot roughly sweeps through the entire bath, which can cause disturbances to the process and may still require manual human intervention when part of the dross is left behind, or the robot gets stuck and cannot clean certain areas in the bath. We aim to improve the traditional approach with the teleoperation approach using haptic assistance that we present and study in this work. By carefully designing a teleoperation system, we could improve the process and reduce the need for this manual and potentially hazardous labor.

Dross removal is a complex, multi-stage operation. First, the dross needs to be collected in an easily reachable place somewhere on the surface of the bath. The motion for this somewhat resembles a wiping or scraping task. After this, the dross needs to be scooped out of the bath which requires careful orientation and placement of the scoop. Now the collected dross needs to be carefully transported to the dross collection bins to not lose the just collected dross. Finally, the dross needs to be dumped into the bin. This once again requires careful orientation and placement of the scoop such that the dross does not fall outside of the bin. Throughout this entire procedure, the operator has to interact with the environment, being careful not to cause any damage with collisions (especially with the galvanized steel strip). Additionally, the operator needs to cause as little disturbance to the bath as possible as this will deteriorate the quality of the zinc coating. This requires careful and accurate movements, especially when directly interacting with the bath. These individual stages generalize well to a range of other tasks related to visuomotor coordination which makes this task an interesting use case.

**FIGURE 1:**
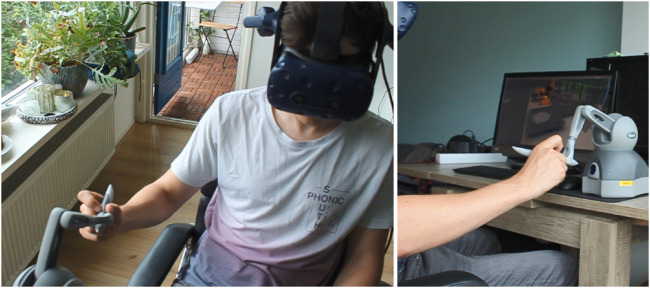
The system used in the experiment setup consisted of the stylus of the Touch Haptic Device and HTC Vive Head-Mounted Display.

Figure 1 shows the main hardware used in the experiment. We used the Geomagic Touch Haptic Device (by 3D Systems) to command the remote robot motion and provide the operator with haptic feedback. Additionally, we used a regular 23 desktop monitor and an HTC Vive HMD device for providing visual feedback and cues in virtual reality (VR).

### 2.2 Design of virtual environment

**FIGURE 2:**
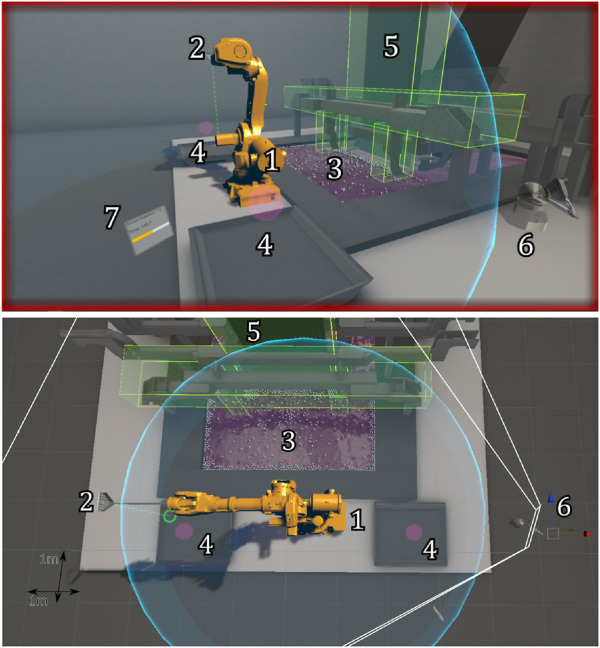
An overview of the virtual environment. The white grid in the bottom figure has a 1 m × 1 m spacing and is shown here only for scale reference. The white lines originating from point 6 show a camera view from which the participant viewed the environment (resulting in the view in the top figure). In other words, both views are the same, however, the view from the monitor is static, whereas with the HMD the view can be altered. When using the HMD, participants can deviate from this view by moving/rotating their heads. Important objects are highlighted by numbers. 1) The controlled simulated industrial robot (robot model: ABB IRB 6640). 2) The scoop that is attached to the robot. 3) The zinc bath containing the dross particles. 4) The bins where the dross is deposited into. 5) The steel strip running through the bath. 6) The virtual representation of the Touch Haptic Device (this object was not visible when viewing the scene on the desktop monitor, as its physical version can be seen in real life). 7) A window showing how much time has passed (text reads as “Current Repetition 1/2, Time: 149.7”). When using the desktop monitor, this same window is shown in the top left corner of the screen.

The virtual environment was built in Unity Game Engine and its layout was designed to be a realistic representation of the dross removal use case. Virtual models of the environment and the robot were imported into the scene and physics colliders were assigned so that the different components could interact with each other. The dross particles were made by using the Obi Fluid Unity plugin and were modified to accurately resemble actual dross behavior inside the bath. We placed a total of 750 dross particles in the bath. This number was chosen so that a skilled operator was able to remove about half the dross within the given time limit. This challenged operators to remove the dross from all areas in the bath, without running out of dross before reaching the time limit and thus preventing ceiling effects in the resulting data. The full environment is shown in Figure 2, which shows the participant’s point of view, and a top view of the virtual environment.

Due to the difference in the workspace size between the haptic device (maximum reach of 0.16 m for Geomagic Touch) and the remote robot (maximum reach of 3.2 m for ABB IRB 6640), we used a scaling factor in the position commands. Additionally, the participants could temporarily decouple and re-couple the haptic device to re-index the workspace and to achieve workspace spanning (Conti and Khatib, 2005; Peternel et al., 2020).

We calculated the interaction force between the slave robot and the virtual environment by using a virtual spring-damper system. This force was then generated on the haptic device to provide the human operator with haptic feedback.

**FIGURE 3:**
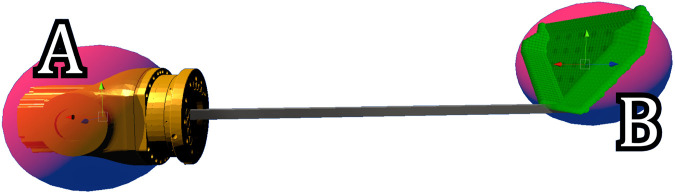
The scoop that’s attached to the simulated industrial robot. The two red spheres show the locations for which the VFs are activated, where **(A)** is the wrist joint and **(B)** is the scoop. The green volume, consisting of small spheres near location **(B)** represents the evenly spaced voxels that were used to approximate the submerged volume of the scoop. In this example, the entirety of the “bucket” part of the scoop is covered by green voxels (over 1,000 small spheres) in high volume.

We supplied HA to the operator in the form of guidance virtual fixtures (GVFs) through the haptic device. We applied guidance forces only in the key areas to aid in difficult parts of the task and they did not influence the feedback elsewhere. The GVFs above the dross bins applied a force so that the scoop (point B in Figure 3) was pulled towards a point located above the center of the dross deposit bin (e.g., (Payandeh and Stanisic, 2002)). This force helped in keeping the scoop centered above the bin when depositing the dross. The guidance force was designed as:

$$F_{VF}=\left\{\begin{array}{cc} k\left(d-d_{0}\cdot \hat{d}\right)\left(1-\frac{\|d\|-d_{t}}{d_{g}}\right),\; & \text{if}\;d_{t}<\|d\|<d_{t}+d_{g}\\ k\left(d-d_{0}\cdot \hat{d}\right),\; & \text{if}\;\|d\|\leq d_{t}\\ 0,\; & \text{otherwise}, \end{array}\right.$$ (1)

where d is the distance between the tip of the scoop and the guidance point above the dross deposit bin, $\hat{d}$ is its unit vector, k is the spring stiffness, $d_{0}$ is the equilibrium of the spring, $d_{t}$ is the distance at which the guidance force is triggered, and $d_{g}$ is the gradient distance, which allowed the force to gradually increase up to the force applied at $d_{t}$ . This was done so that the operator did not experience a sudden shock when the guidance force jumped to its maximum value. Note that if $d_{g}=0$ , the range $d_{t}<\|d\|<d_{t}+d_{g}$ does not exist.

Forbidden region virtual fixtures (FRVFs) acted as an artificial force field, pushing the operator away from dangerous areas (e.g., (Abbott, 2005)). The magnitude of this force was calculated by the sum of Equation 1 calculated for the wrist and the tip of the scoop (i.e., points A and B in Figure 3, respectively). In this case, d is the distance between the wrist or the scoop and the boundary of the forbidden region. The distances in Table 1 were chosen such that with an average movement speed, participants would still be able to react to the cues in time while minimizing the activation distance such that the cues distract as little as possible when they are not relevant.

**TABLE 1:** The parameters used for the guidance forces of the VFs.

	k (N/m)	$d_{0}$ (m)	$d_{t}$ (m)	$d_{g}$ (m)
Bin GVF	2.75	0	0.2	0.1
FRVFs	4.5	0.3	0.3	0

The GVF in the bath acted as a buoyancy, pushing the scoop out of the bath and helping to keep the scoop at the correct height. The magnitude of this force was scaled by the volume of the scoop that was submerged in the bath. This volume was approximated with the use of voxels (volumetric pixels). At each sample time, an array of (roughly 1,200) evenly-spaced voxels (the green balls in Figure 3) was checked to find the fraction of voxels below the bath surface. The magnitude of the guidance force was equal to this submerged fraction multiplied by the constant α=1.5 . This scaling was chosen such that the guidance force was clearly noticeable without disturbing the operator more than needed. Similar to the guidance force, the disturbance also scaled with the submerged volume. In this way, the operator received (indirect) feedback about the bath disturbance and was motivated to keep this disturbance to a minimum. Contrary to the disturbance measure (Section 3.3), the guidance force was not multiplied with the scoop velocity to keep the feedback easy to interpret.

### 2.3 Design of visual feedback

Figure 2 shows the scene view with the designed visual cues. The first of these visual cues is a purely spatial cue to improve the operator’s spatial and situational awareness. This cue showed the position of the scoop’s tip, projected in the direction of gravity. A semi-transparent green dashed line was drawn towards the first surface it intersected. At the end of this line, a green circle with a radius of 0.025 m was drawn to clearly show the position of the scoop (in the x-y plane), and what lies underneath it.

The remainder of visual cues are related to the different types of forces that could be experienced. More specifically, they informed the operator where the force originated from and visualized the magnitude and direction of that force. Each force type is visually represented by specialized visual cues that are explained below.

#### 2.3.1 Collision force design

**FIGURE 4:**
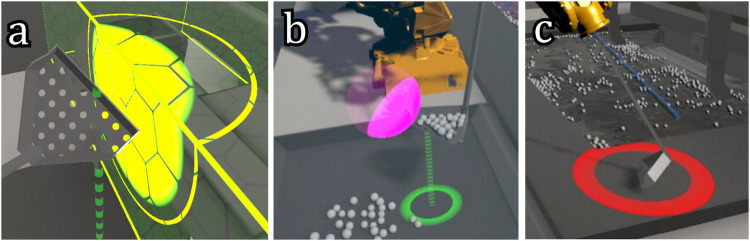
The designed visual cues that represent different sources of feedback forces and their magnitudes as explained in Section 2.3. Picture **(A)** shows the FRVF (forbidden region virtual fixture) force cue, designed to guide operators away from dangerous areas. Picture **(B)** illustrates the GVF (guidance virtual fixture) force cue above the dross deposit bins, to help the operator with the accurate placement of the scoop above the bin. Picture **(C)** depicts the collision force cue indicated by a red ring that warns the operator of current collisions.

If the scoop collided with another object, a red ring would appear at the point of collision (Figure 4C). The radius of this ring was proportional to the magnitude of the collision force, with a minimum of 0.15 m. The scaling was such that the radius was 1.1 m at the maximum collision force (Section 3.1). Besides informing the operators about if and where they were colliding, this cue also provides information about the amount of force they were applying to the environment when colliding (Horeman et al., 2014).

#### 2.3.2 Virtual fixture design

Figure 2 shows all VFs in the environment. The semi-transparent yellow boxes show the boundaries of the FRVFs, while the semi-transparent purple areas show the GVFs. This cue was partially inspired by the work in Giulietti et al. (2016), where the forbidden regions were visualized to improve the safety of piloting a remotely piloted vehicle.

Furthermore, if the guidance force of a VF is greater than zero, an additional visual cue appears at the location from which this guidance force originates. For the FRVFs and the GVF in the bath, this cue consisted of a bright yellow circle, overlaid onto the VF visualization, as shown in Figure 4A. The radius of the circle in the center was dependent on the current magnitude of the guidance force. The outer ring represented the maximum guidance force possible for this VF. If this maximum guidance force was reached and the whole circle was filled, it started to blink to warn the operator. In this way, operators were informed about where the force came from and what its magnitude was. Additionally, operators were (indirectly) informed of how close they were to the boundary of the VF. This visual cue was in part inspired by the work in Nakazawa et al. (2016), where the proximity to a cone-shaped boundary in a deep and narrow surgery task was visualized by using a semicircle on the edges of the screen. Additionally, visualizing the operational boundaries and the controlled system’s current relation to them is beneficial in automotive (Vreugdenhil et al., 2019) and aviation domains (Borst et al., 2013). Therefore, we designed a similar cue that can be suitable for the teleoperation of a simulated industrial robot (Figure 2).

The GVF above the bins was different, as it consisted of a single point. The GVF itself was visualized by a purple sphere, centered onto the guidance point that it represented (Figure 4B). The radius of this sphere was the trigger distance described in Section 2.2. Unlike the other two VF cues, this cue did not show the magnitude of the applied force but simply lit up an area around the point on the scoop on which the GVF acted. This area lit up slightly before the scoop got close enough to activate it so that the guidance force and its direction could be anticipated by the operator.

#### 2.3.3 Workspace feedback design

During teleoperation, the operator has to take the workspace of the controlled robot into account. If its limits are reached, the force feedback behaves the same as when a collision with the environment occurs, resulting in a haptic force pulling the operator back into the workspace. Therefore, the robot workspace is visualized to inform the operator of this limit (as shown in Figure 2). The visualization of this haptic force behaves in the same way as described for the FRVFs and GVF in the bath discussed in the previous section, and the design choices here follow the same rationale.

## 3 Experiment methods

Sixteen participants^1^ (4 females) between 20 and 52 years old (M=24.8,SD=7.6) volunteered for the experiment. All participants gave their informed consent prior to the experiment. The setup and experiments were approved by the Human Research Ethics Committee of the Delft University of Technology.

### 3.1 Task description

The participants were instructed to remove as much dross as they could within the time limit of 5 minutes while ensuring a safe operation. An overview of the virtual environment is shown in Figure 2. The dross was removed by controlling the simulated industrial robot using the Geomagic Touch haptic device. The control point was set to the wrist joint of the scoop (as if the operator was holding the scoop at the end of a rod). Using the scoop, the dross particles were removed from the zinc bath and deposited into one of the two dross deposit bins.

The participants were instructed to cause as little disturbance to the bath as they could. This is important, as such disturbances lead to a deterioration of the quality of the coating applied in the galvanization process. The participants were informed to minimize this disturbance by minimizing the submerged volume of the scoop, and the velocity with which the scoop moved through the bath. Furthermore, the participants were instructed to try to avoid collisions and minimize the collision force when collisions do occur. Lastly, two safety-related boundary conditions (BCs) were defined, and if they were violated, the current task session was stopped and considered a failed session. During the experiment, the virtual environment indicated the failure of a session by a text message. Prior to the experiments, participants were instructed that avoiding such failed sessions was their most important objective. These BCs are explained below.

#### 3.1.1 Maximum collision force

Large collision forces can damage the robot and its surroundings. For this reason, a maximum collision force of 40 N was set. The value of this maximum was set in such a way that it was not easily exceeded, as long as the robot was operated in a slow and controlled manner.

#### 3.1.2 Collision with the steel strip

If the robot collides with the steel strip in a continuous galvanizing line (CGL), it can damage the strip and potentially result in a shutdown of the entire galvanizing line, resulting in significant financial losses. In the experiment, touching the steel strip stopped the current task session. While the HA helped to push the operators away from the steel strip, they might have been motivated to approach it, as there were dross particles nearby.

### 3.2 Experiment design

**FIGURE 5:**
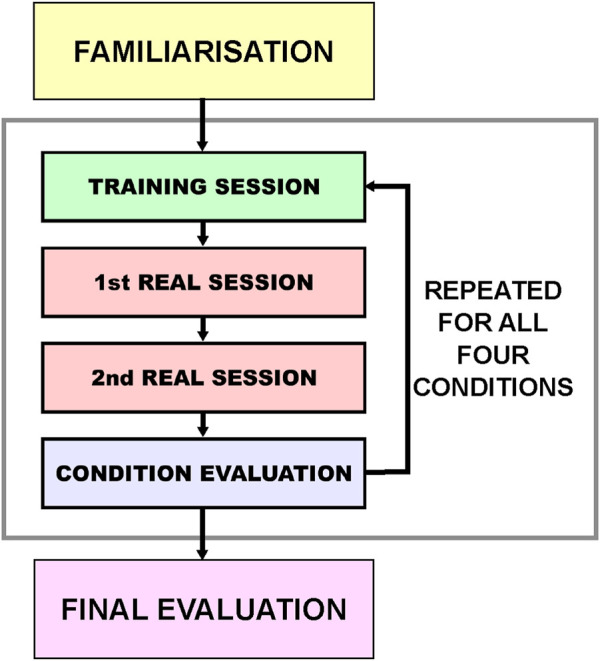
Design of experiment protocol. The experiment started with a familiarisation where the participants got familiar with the experiment setup and the task. The main part of the experiment involved four conditions presented in a counter-balanced order to each participant. Each condition had one training session and two real sessions (repetitions), where each of them lasted 5 min, thus the number of scooping actions (trials) varied. After all sessions in each condition, a subjective evaluation was conducted by the participants to assess that condition. At the end after all conditions, a final evaluation questionnaire was provided to assess the preference over the four conditions.

The experiment protocol is outlined in Figure 5. The effect of the two independent variables was evaluated in a counter-balanced 2 (Display: Monitor vs. HMD) x 2 (Cues: With vs. Without) within-subjects design. In other words, there was a total of four experimental conditions, which are abbreviated for ease of reference as:

• MN: Monitor display, and No visual cues
• MC: Monitor display, and with visual Cues
• HN: HMD, and No visual cues
• HC: HMD, and with visual Cues

These conditions were ordered according to an incomplete counter-balanced measures design. The participants first received a training session of about 10 min, during which they were able to toggle the presence of each of the haptic and visual cues and switch between displays.

Once the participants were confident in their ability to use the interface, they started with the first condition. The current session only started when the haptic device was first connected to the robot. For each condition, a practice session of 5 minutes was performed, in which participants familiarized themselves with the current interface and practiced their scooping strategy. After this, the participants performed two real sessions (repetitions), which together were used for the calculations of the dependent measures of that particular experimental condition. If one of the sessions failed (due to violating one of the BCs), only that session was stopped.

There were two real sessions (repetitions) per condition, which resulted in eight sessions performed by each participant. The number of scoops each participant performed during each session defined the number of trials collected during that session, which varied since the time of each session was fixed to 5 min (or less if a failure occurred). On average, participants managed to perform around 28 trials per session (see Figure 9-left in the results for exact numbers).

### 3.3 Dependent measures

To calculate the dependent measures, we combined the data of two sessions performed for each condition. If a session was shortened because the BCs were violated, the data for this session was still included, but that session simply lasted less time (i.e., less opportunity to remove dross).

We defined the performance metrics as:

• Percentage of Dross Removed (%). Dross is considered removed only when the particles have been deposited into one of the two bins. The total amount of dross removed over the two sessions is divided by the total amount of dross present in those sessions (1,500 particles). This is used as the main performance measure.
• Average Bath Disturbance (volume fraction ⋅m/s ). The bath disturbance is approximated by multiplying the fraction of the submerged volume (as used by the GVF in the bath, described in Section 2.2) with the velocity of the end of the scoop (point B, shown in Figure 3). The resulting value is averaged over time. The participants were instructed that for this task, it is important to minimize this measure by submerging the scoop only as much as necessary, especially while making large motions through the bath. The resulting scores are used as a measure of the performance, as a high disturbance will lead to unsatisfactory results in the galvanizing process, regardless of the amount of dross that is removed. Additionally, this measure indicates the accuracy with which the task can be performed, as minimizing this measure requires operators to maintain an accurate distance to the liquid level.

We defined the safety metrics as:

• Number of sessions failed. Sessions fail and are stopped if the BCs are violated. Besides counting this number, the failure of a session is reflected in the performance measures as well, as this failure leads to participants having less time to perform the operation.
• Peak collision Force (N). The participants are instructed to minimize the collision forces. The peak collision force indicates how close they got to failing the session as a result of collisions with the environment. The peak collision force is the maximum exerted force over both sessions. This value is used as the main safety measure.
• Minimum distance to steel strip (m). Collision with the steel strip results in significant financial losses. For this reason, if this occurred during the experiment, that session fails and is stopped immediately. As such, getting close to the steel strip is risky, and the minimum distance to the steel strip indicates the amount of risk of the operation.

We defined the user acceptance metrics as:

• Van der Laan Questionnaire. The user acceptance of the interface was assessed using the Van der Laan acceptance questionnaire (Van Der Laan et al., 1997), where the participants reported usefulness and satisfaction scores after each experimental condition. The participants were specifically instructed to rate the interface design as a whole (the *cues*, the *display*, the *haptic interface*, and the *haptic feedback and guidance*).
• Preferred Condition. In a final questionnaire, the participants were asked which experiment condition they preferred. This questionnaire also consisted of four questions. First, they were asked what was difficult about this task (Q1). Then they were asked what experimental conditions they liked the most (Q2), and why (Q3). Lastly, they were asked if they had any additional remarks (Q4).

Furthermore, we identified four *supplementary metrics* for a supplementary analysis to help us gain additional insights. These metrics are defined as:

• Peak Scoop Submergence Rate (voxels/s) . The rate at which the scoop is submerged into the bath is calculated by evaluating the number of voxels (see Figure 3) that go into the bath for each timestep. The peak value for each session was evaluated to gain additional insight into the disturbance caused to the bath. As mentioned above, participants are asked to cause minimal bath disturbance during the task and therefore should aim to minimize this value.
• Average Scoop Velocity (m/s). The average scoop velocity was calculated by averaging the velocity of the tip of the scoop over time for each session. This metric was evaluated as a means to gain insight into how well participants were able to move the robot through space under different conditions. The higher average velocity also enables more trials in the time-limited session.
• Number of Scoops. The number of scoops for each session is calculated by finding the timestamps at which changes took place in the amount of dross in the bins and the bath. Using these timestamps, the task can be divided into the sub-tasks of dross scooping and dross dumping, giving us the total number of scoops (trials) for each session.
• Average Scoop Size (particles/scoop). In order to take dross particles out of the bath, it is critical to appropriately orient the scoop during the whole movement to maximize the dross collection. Therefore, we evaluate the average scoop size for each session to gain insight into how well participants were able to control the orientation of the scoop under different conditions. The average scoop size is found by dividing the total amount of dross particles collected by the number of scoops (trials) for each session.

## 4 Results

In this section, the experiment results are presented in accordance with the dependent measures as explained in Section 3.3. In the analysis, we set the statistical significance threshold α=0.05 . Table 2 shows the means and standard deviations for all dependent measures, along with the results of the two-way RM ANOVA. The most important results are discussed in the text in this section, though for the full details of the statistical analysis, the reader is referred to Table 2. Since only one measure required *post hoc* pairwise analysis (satisfaction score), its results are presented in the text in Section 4.3.

**TABLE 2:** Means (M), standard deviations (SD), and results of the two-way repeated measures ANOVA for each dependent measure.

Measures		Conditions				RM ANOVA, F (1,15)		
		MN	MC	HN	HC	Cues	Display	Interaction
Performance								
Percentage Dross Removed (%)	M SD	25.28 14.89	25.36 11.54	30.12 14.53	31.73 12.46	p=0.7425 F=0.11	p=0.0072 F=9.68	p=0.7174 F=0.13
Average Bath Disturbance (volume fraction ⋅m/s )	M SD	$2.21\cdot 10^{-2}$ $1.68\cdot 10^{-2}$	$1.72\cdot 10^{-2}$ $0.96\cdot 10^{-2}$	$1.96\cdot 10^{-2}$ $1.39\cdot 10^{-2}$	$1.73\cdot 10^{-2}$ $1.03\cdot 10^{-2}$	p=0.1043 F=2.99	p=0.1882 F=1.90	p=0.3589 F=0.95
Safety								
Number of failed sessions (Count)		3	0	3	0			
Peak collision force (N)	M SD	26.40 7.59	22.62 6.61	27.89 9.13	19.89 6.30	p=0.0003 F=22.19	p=0.6116 F=0.27	p=0.2917 F=1.19
Min. distance to steel strip (m)	M SD	0.57 0.20	0.71 0.27	0.64 0.30	0.63 0.23	p=0.1399 F=2.43	p=0.3518 F=0.92	p=0.5342 F=0.41
User Acceptance								
Preferred Condition (Count)		0	2	2	12			
Satisfaction score* (−2,2)	M SD	0.28 0.52	1.13 0.72	0.98 0.49	1.25 0.69	p=0.0012 F=15.84	p=0.0010 F=16.76	p=0.0405 F=5.03
Usefulness score* (−2,2)	M SD	−0.09 0.76	0.50 0.56	0.70 0.48	0.81 0.58	p=0.0100 F=8.68	p<0.0001 F=42.61	p=0.1316 F=2.54
Supplementary Analysis								
Peak scoop submergence rate* (voxels/s)	M SD	44.27 20.16	33.87 18.62	36.84 20.34	25.22 18.25	p=0.0200 F=6.78	p=0.0595 F=4.16	p=0.8969 F=0.02
Average Scoop Velocity (m/s)	M SD	0.45 0.08	0.44 0.09	0.47 0.09	0.45 0.08	p=0.1145 F=2.81	p=0.0304 F=5.71	p=0.7856 F=0.08
Number of scoops* (Count)	M SD	24.50 11.28	25.88 7.30	26.88 10.01	28.25 6.75	p=0.6315 F=0.24	p=0.1175 F=2.76	p=0.8286 F=0.05
Average scoop size (particles/scoop)	M SD	14.34 5.50	14.75 4.77	16.75 4.60	16.95 4.37	p=0.7081 F=0.15	p=0.0004 F=21.11	p=0.8918 F=0.019

* Shapiro-Wilk test violated, data transformed using aligned rank transformation (Wobbrock et al., 2011). Significant effects (p≤0.05) are printed in bold. Condition abbreviations are described in Section 3.2.

### 4.1 Performance

**FIGURE 6:**
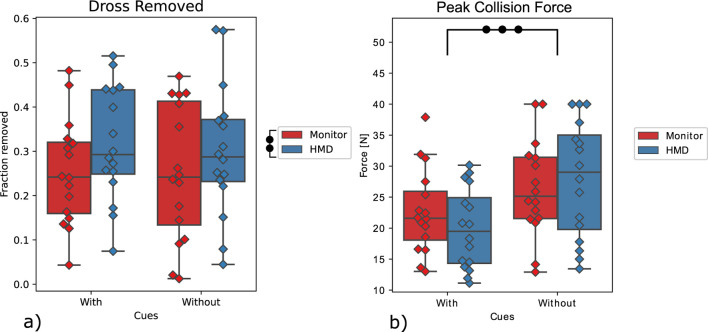
**(A)** Percentage of dross removed (main performance measure), **(B)** Peak collision force (main safety measure), as displayed on the vertical axes. The presence of visual cues is indicated on the horizontal axes. The colors depict the display method. The box-whisker plots depict the median (horizontal line inside the box), interquartile range (box) and extreme values (whisker) for a given variable. The diamonds indicate the individual values of all participants. The marks denote significance, where • p≤0.05 , •• p≤0.01 , ••• p≤0.001 .

For the percentage of dross removed, there was a significant main effect of the *display*, F(1,15)=9.68 , p=0.007 (Table 2; Figure 6A), where the HMD method achieved a significantly higher percentage of removed dross compared to the desktop monitor. However, there was no statistically significant main effect of the *cues* (p=0.74) , and no statistically significant interaction effect (p=0.72) was found for this measure. Furthermore, no significant effects were found for the average bath disturbance (Table 2). However, it is noteworthy that the means of the bath disturbance show a slight, but insignificant decrease with the use of the visual cues method (p=0.10) .

### 4.2 Safety

Out of the combined total of sessions of all participants and all conditions (128 sessions), a total of six sessions failed due to a violation of one of the BCs. All failed sessions occurred during the conditions without visual cues, where three occurred with the HMD (HN) and three occurred with the desktop monitor (MN). Only in one of these sessions, the failure was caused by a collision with the steel strip, which occurred during the MN condition.

For the peak collision force, a significant effect of the *cues* was found F(1,15)=22.19 , p=.0003 (Table 2; Figure 6B), where the peak collision force was significantly lower with visual cues compared to without them. However, no such effect was found for the type of *display* (p=0.61) , and no significant interaction effect was found (p=0.29) . Furthermore, no significant effects were found for the minimum distance to the steel strip (Table 2).

### 4.3 User acceptance

**FIGURE 7:**
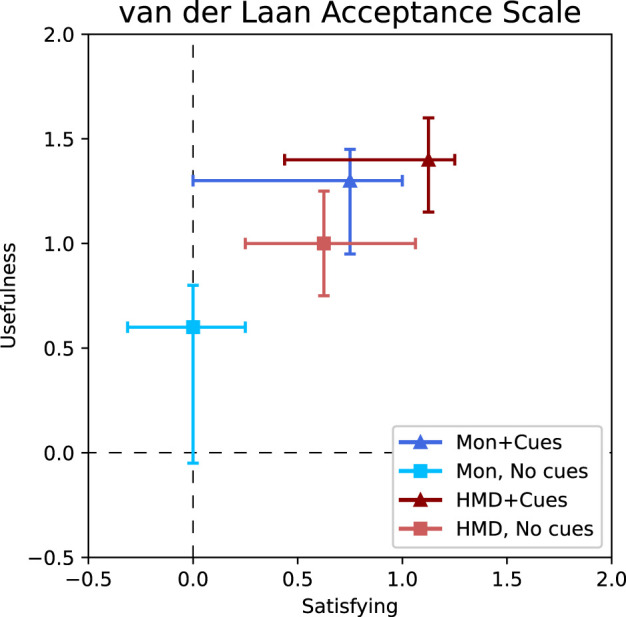
Van der Laan acceptance scores (Van Der Laan et al., 1997); on the horizontal axis is the satisfying dimension and on the vertical axis is the usefulness dimension. Scores are in the range of (−2, 2). The center points are the median values, and the colored lines show 25% percentile ranges. Additional details are stated in Table 2.

Out of the 16 participants, 12 participants preferred the HC condition (Section 3.2). The other four participants were evenly divided over the MC and HN conditions. Furthermore, both the HMD and the visual cues significantly improved both the satisfaction and the usefulness score (Table 2; Figure 7). However, the interaction effect of the satisfaction score was also significant, F(1,15)=5.03,p=0.04 . This means that for the satisfaction score, the effect of the display mode depends on the effect of visual cues and vice versa. The pairwise *post hoc* comparison revealed a significant effect of the visual cues, only when the HMD was not used (MC-MN: p=0.0048 , HC-HN: p=0.071 ). Likewise, the effect of the HMD is only significant when the visual cues are not present (MN-HN: p=0.0015 , MC-HC: p=0.173 ). Scores for the individual questions for the four conditions are shown in Table 3.

**TABLE 3:** Scores for the individual questions of van der Laan questionnaire (Van Der Laan et al., 1997) for the four conditions. The last row indicates how many participants preferred each condition.

Questions		Conditions			
		MN	MC	HN	HC
Usefulness					
1: Useless … Useful	M	0.31	1.25	1.25	1.19
	SD	1.10	0.56	0.56	0.95
3: Bad … Good	M	0.50	1.06	0.81	1.00
	SD	0.87	0.56	0.63	0.71
5: Superfluous … Effective	M	0.06	1.00	1.06	1.50
	SD	1.09	0.61	0.75	0.61
7: Worthless … Assisting	M	0.13	1.13	0.94	1.25
	SD	0.99	1.05	0.66	0.83
9: Sleep … Alert	M	0.38	1.19	0.81	1.31
	SD	0.93	0.73	1.07	0.58
Satisfying					
2: Unpleasant … Pleasant	M	−0.13	0.50	1.00	0.94
	SD	0.99	0.79	0.50	1.09
4: Annoying … Nice	M	0.38	0.56	0.56	0.75
	SD	0.93	0.86	1.00	0.83
6: Irritating … Likeable	M	−0.19	0.38	0.75	0.56
	SD	0.73	0.99	0.56	0.79
8: Undesirable … Desirable	M	−0.44	0.56	0.50	1.00
	SD	0.70	0.86	0.71	0.71
Final Questionnaire					
Preferred Condition (total count)		0	2	2	12

### 4.4 Supplementary analysis

**FIGURE 8:**
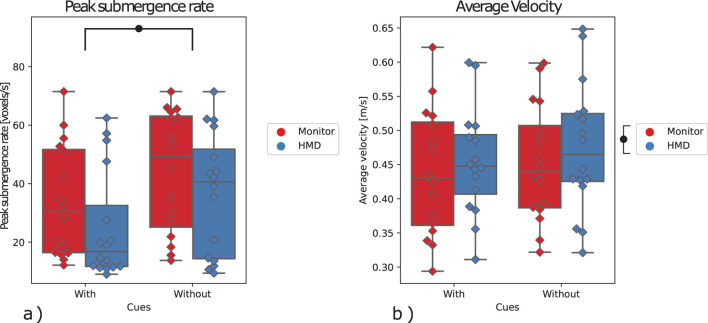
**(A)** Peak scoop submergence rate, **(B)** Average scoop velocity. The marks denote significance, where • p≤0.05 , •• p≤0.01 , ••• p≤0.001 .

For the peak scoop submergence rate, a significant effect was found for the visual *cues*, with F(1,15)=7.03 , p=.018 (Table 2; Figure 8A). Here, this peak was found to be significantly lower with the addition of visual cues (similar to the peak collision force in Section 4.2). No significant effects were found for the factor of the display or the interaction between the two factors.

**FIGURE 9:**
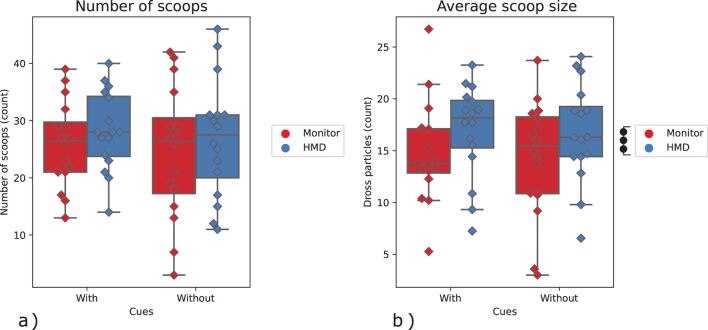
**(A)** Number of scoops taken, **(B)** Average scoop size (amount of dross particles per scoop). The marks denote significance, where • p≤0.05 , •• p≤0.01 , ••• p≤0.001 .

The average scoop size was also found to be significantly higher with the use of an HMD as compared to that of a monitor F(1,15)=21.11 , p=.0004 (Table 2; Figure 8B). Similarly, The average scoop velocity was found to be significantly higher with the use of the HMD F(1,15)=5.71 , p=.030 (Table 2; Figure 9B). No interaction effects or effects of the visual cues factor were found. Furthermore, no significant effects were found on the number of scoops taken for each session (Table 2; Figure 9A).

## 5 Discussion

In the overview, most of the hypotheses were confirmed (H2, H3, H4, H5, H6) and one was rejected (H1). H1 was rejected as the performance was not improved by the proposed visual cues method, however, H2 was confirmed as user acceptance was improved. The safety was improved to some degree, thus confirming H3. H4 was confirmed since the HMD method significantly improved the performance. H5 was also confirmed since user acceptance improved by the HMD method. H6, related to the absence of an interaction effect, was confirmed for all measures, except for the satisfaction score. This suggests that, as hypothesized, the two visual feedback methods have some unique benefits, and (when an effect was found) they improved the operation regardless of the presence the other method. The exception to this is discussed below, in Section 5.3.

### 5.1 Effects of visual cues on safety

The visual cues method was found to significantly improve the safety of the operation, such that the peak collision force was significantly lower, confirming H3. This indicates that visual cues are important in improving the safety of the operation. This statement is supported by the fact that none of the six failed sessions occurred when the visual cues were present. Similar improvements in safety have been found in previous research in aviation (Borst et al., 2013; Ho et al., 2018), automotive (Vreugdenhil et al., 2019), and (narrow) surgery (Nakazawa et al., 2016) domains. A likely reason why these improvements can be found is that the participants were more aware of which forces were present and had a better understanding of where those forces came from (which was a common statement in the comments of the final questionnaire).

That being said, this result is not reflected in the minimum distance to the steel strip, which opposes the results from the above-mentioned research that examined different domains. A logical reason for this is that the tasks considered in those domains provided no direct benefits of getting closer to the forbidden regions and mostly induced risk (e.g., moving a vehicle into an obstacle). In contrast, in the examined dross-removal task, getting close to the forbidden regions (even penetrating them on some occasions) allows the operator to collect more of the dross particles, increasing their performance scores. The final questionnaire revealed that the participants felt that they were more aware of the perceived dangers with the presence of visual cues. This is likely the reason why the participants did not keep a greater distance to the steel strip and were willing to accept the risks in exchange for performance.

### 5.2 Effects on performance

The task performance was not significantly affected by the addition of visual cues, thus rejecting H1. The study in Borst et al. (2013) suggested that visual cues complementing HA can help operators to more accurately follow an optimal trajectory. However, it should be noted that the examined dross-removal task was much more complex than a simple trajectory following. Nonetheless, we expected that the additional visual cues would similarly result in a reduction of the average bath disturbance. Although a slight decrease was found, the effect was not significant. This suggests that the visual cues did not help the participants in recognizing how much disturbance they were causing to the bath.

Additionally, we found relatively large standard deviations for these datasets (Table 2). This variability could be explained by the fact that the participants did not receive any direct feedback about how much disturbance they had caused for each session (as opposed to the percentage of dross removed). Most likely, the participants were unaware of the significance of this aspect of the experiment, despite it being explicitly stated during the training session. The absence of an effect for the percentage of dross removed could also be caused by the participants having a better sense of danger, and as a result being more careful in their navigation around the environment (Section 5.1). This could prevent the predicted increase in performance, even though operators are supplied with additional spatial cues.

Improvement of performance with the use of the HMD, as predicted by H4, was confirmed since a significant effect was found for the main performance measure (percentage of dross removed). This indicates that the use of the HMD helps operators control the robot in a way that more dross can be removed. This result is also in line with the previous research, in which performance enhancements were found when depth perception is an important factor (Liu et al., 2017), and when the interface is not intuitive (Whitney et al., 2020). Future work could entail a study of how exactly depth perception affects task performance in the teleoperation of industrial robotic arms. However, this would require a separate study where the depth perception factor would be isolated from other influencing factors. For example, the study in Luo et al. (2021) specifically investigated the stereoscopic factor during the use of HMD for teleoperation of a vehicle but did not involve any additional factors that we examine here, i.e., haptic feedback or visual cues.

However, such an effect was not found in the results of the average bath disturbance. This indicates that even though the HMD helps operators in positioning and orienting the scoop, it did not help in accurately keeping the scoop at just the right height to minimize bath disturbance. Additionally, as explained above, the large variability in these results could have been caused by a lack of clear feedback regarding the amount of disturbance caused.

While we examined four main conditions in the current study, a future study could also use other conditions for the comparison. For example, the operator performance between using force-feedback-related visual cues and visual cues without force feedback could be compared.

### 5.3 Effects on user acceptance

As hypothesized, both the visual cues method (H2), as well as the HMD method (H5), resulted in a significantly improved user acceptance, for both the satisfaction and the usefulness score. This was further supported by the scores of the preferred condition, as seen in Table 2. This result is in accordance with previous studies from different domains, in which similar improvements in user acceptance were found as a result of using visual cues (Ho et al., 2018; Borst et al., 2013; Nakazawa et al., 2016) and using an HMD (Whitney et al., 2020). Our study shows that these results can be extended to HA teleoperation of robotic arms.

We did not find a significant interaction effect for most of our variables, thus confirming H6. However, we did find an interaction effect on the satisfaction score. The pair-wise comparison revealed that when one of the methods was already present, the participants did not perceive the interface as more satisfying with the addition of the other method. A possible explanation for this is that visual cues can obstruct the line of sight in some cases (according to the comments from two participants). This is in analogy with the work in Ho et al. (2018), where it was found that in some situations the visual cues would cause clutter, resulting in a decrease in user acceptance and even decreased performance. While the addition of the other feedback method did not impair performance or safety, it may have introduced additional stimuli and cognitive load that led to decreased satisfaction. This highlights the need for careful design and presentation of visual cues to enhance user satisfaction without compromising usability. Alternatively, the absence of an interaction effect may be attributed to either visual cues or the display method independently providing sufficient additional depth information. Therefore, the primary benefit was likely already achieved with either method alone, and the incremental advantages of incorporating both (e.g., visualization of interaction forces) were not substantial enough to produce a noticeable difference in user satisfaction score.

### 5.4 Supplementary analysis

Similar to the peak collision force, the peak submergence rate decreased significantly with the implementation of the visual cues. This indicates that visual cues about the interaction forces help with reducing the disturbance to the bath, and thus improve the aspects related to physical interaction.

The results about average velocity suggest that with the HMD, participants had an easier time navigating the environment, and were able to move slightly (but significantly) faster as a result. Interestingly, this did not result in participants being able to perform more scoops (trials) per session as a result. However, considering higher scoop sizes and more dross being removed while using HMD, it implies that the participants were able to maximize velocity in less complex parts of the task (e.g., approach) in order to use the gained time in more complex parts (e.g., collection).

While visual cues about the interaction forces improved the peak submergence rate, they did not help to improve the average velocity, number of scopes, and average scoop size. This is likely due to the peak submergence rate being dominated by physical interaction, while the other three metrics are dominated by positions/orienting actions.

### 5.5 Limitations

The key limitation of the current study is that the remote robot and environment were simulated in a virtual environment. There might be some differences between operating a simulated robot in a virtual environment and operating a real robot in the actual environment. We expect that the main difference between simulation and real setup would be transmission delays in the teleoperation loop. This could further increase the task complexity in the sense that the operator would have to better predict the actions in advance to counter the delayed feedback. Additional visual cues about interaction forces could help in making such predictions. However, this should be explored in a future study. Nevertheless, we believe that the results from a simulated robot in a virtual environment are still valuable as they can be used for training novice operators how to perform complex tasks such as dross removal before they can operate a real robot in an actual environment. Setups using a simulated remote robot in a virtual environment are important platforms for operator training (Jourdes et al., 2022; Sunesson et al., 2023; Kuitert et al., 2023).

The results have quite some variability in the data. This could potentially be attributed to the diversity of participants in terms of experience in teleoperation and gaming. Furthermore, the teleoperated dross removal task is quite difficult in general, and according to the informal feedback from the participants, the difficulty between the stages of the task varied (i.e., scooping was harder than depositing and reaching), which could have further contributed to the variability. This could have led to an inconsistent participant response, as some may not have been able to fully leverage the feedback methods due to the task’s difficulty. A future study should focus on the identification of the varying complexity of different stages of the task, as well as the influence of various prior experiences.

## 6 Conclusion

This study takes an important step towards gaining a better understanding of the importance of visual feedback design in HA teleoperation of industrial robotic arms in manufacturing tasks. Furthermore, it adds to the limited amount of work that has investigated this interaction and provides new evidence to support the idea that this synergy is worth exploring further. From the results of this study, the following is concluded:

• The proposed visual cues method improves the operation’s safety and helps prevent excessive forces and collisions with dangerous obstacles. These effects were found regardless of the use of the HMD method.
• The use of the HMD method compared to the use of the desktop monitor increases the main task performance, without compromising the safety and accuracy of the operation.
• The performance improvements caused by the use of the HMD method are present regardless of the use of the visual cues method.
• In general, acceptance improved with the use of either the visual cues method or the HMD method. However, when one of the methods is already present, the use of the other method did not further increase the satisfaction scores.
• HMD helps to improve positioning/orienting aspects within the task, while visual cues about the interaction forces help to improve aspects dominated by physical interaction.

These results indicate that although both methods provide benefits, they do so in different aspects. Task performance mostly benefits from the use of the HMD, whereas safety mostly benefits from the use of the proposed visual cues method. Moreover, the results for user acceptance indicate that, although both methods improve user acceptance, combining them might not cause further improvements. As such, interface designers should be careful to consider the necessity of including each of the proposed visual feedback methods.

## Data Availability

The raw data supporting the conclusions of this article will be made available by the authors, without undue reservation.

## Footnotes

1 Participants were asked how much experience they had with teleoperation. In response, six participants reported having never done it, two have about 1 h of experience, six have about 10 h of experience, and two have about 1 day of the experience. Additionally, the participants were asked about their experience with video gaming. Most participants have more than 10 weeks of experience (12 participants), two participants reported having about 10 weeks of experience and the last two participants reported having about 10 h of gaming experience.
